# Redirection of Metabolic Hydrogen by Inhibiting Methanogenesis in the Rumen Simulation Technique (RUSITEC)

**DOI:** 10.3389/fmicb.2017.00393

**Published:** 2017-03-14

**Authors:** Jessie Guyader, Emilio M. Ungerfeld, Karen A. Beauchemin

**Affiliations:** ^1^Lethbridge Research and Development Centre, Agriculture and Agri-Food CanadaLethbridge, AB, Canada; ^2^Instituto de Investigaciones Agropecuarias INIA CarillancaTemuco, Chile

**Keywords:** fermentation, hydrogen, *in vitro*, methane, microbial biomass, reduced product, volatile fatty acid

## Abstract

A decrease in methanogenesis is expected to improve ruminant performance by allocating rumen metabolic hydrogen ([2H]) to more energy-rendering fermentation pathways for the animal. However, decreases in methane (CH_4_) emissions of up to 30% are not always linked with greater performance. Therefore, the aim of this study was to understand the fate of [2H] when CH_4_ production in the rumen is inhibited by known methanogenesis inhibitors (nitrate, NIT; 3-nitrooxypropanol, NOP; anthraquinone, AQ) in comparison with a control treatment (CON) with the Rumen Simulation Technique (RUSITEC). Measurements started after 1 week adaptation. Substrate disappearance was not modified by methanogenesis inhibitors. Nitrate mostly seemed to decrease [2H] availability by acting as an electron acceptor competing with methanogenesis. As a consequence, NIT decreased CH_4_ production (−75%), dissolved dihydrogen (H_2_) concentration (−30%) and the percentages of reduced volatile fatty acids (butyrate, isobutyrate, valerate, isovalerate, caproate and heptanoate) except propionate, but increased acetate molar percentage, ethanol concentration and the efficiency of microbial nitrogen synthesis (+14%) without affecting gaseous H_2_. Nitrooxypropanol decreased methanogenesis (−75%) while increasing both gaseous and dissolved H_2_ concentrations (+81% and +24%, respectively). Moreover, NOP decreased acetate and isovalerate molar percentages and increased butyrate, valerate, caproate and heptanoate molar percentages as well as n-propanol and ammonium concentrations. Methanogenesis inhibition with AQ (−26%) was associated with higher gaseous H_2_ production (+70%) but lower dissolved H_2_ concentration (−76%), evidencing a lack of relationship between the two H_2_ forms. Anthraquinone increased ammonium concentration, caproate and heptanoate molar percentages but decreased acetate and isobutyrate molar percentages, total microbial nitrogen production and efficiency of microbial protein synthesis (−16%). Overall, NOP and AQ increased the amount of reduced volatile fatty acids, but part of [2H] spared from methanogenesis was lost as gaseous H_2_. Finally, [2H] recovery was similar among CON, NOP and AQ but was largely lower than 100%. Consequently, further studies are required to discover other so far unidentified [2H] sinks for a better understanding of the metabolic pathways involved in [2H] production and utilization.

## Introduction

In the rumen, metabolic hydrogen ([2H]) is released during the fermentation of feed by bacteria, protozoa and fungi. Metabolic hydrogen transfer to different acceptors ensures the continuity of fermentation by re-oxidizing reduced co-factors. Dihydrogen (H_2_) is produced when electrons are transferred to protons in reactions catalyzed by hydrogenases (Hegarty and Gerdes, 1999). Hydrogenase activity can be inhibited by an accumulation of H_2_, with bacterial hydrogenases ([Ni-Fe] hydrogenases) being more sensitive than protozoal hydrogenases ([Fe-Fe] hydrogenases) (Fourmond et al., 2013). Metabolic hydrogen can also be incorporated into other pathways, including propionate, methane (CH_4_) and microbial protein synthesis (Henderson, 1980; Czerkawski, 1986; Asanuma et al., 1999). The flow of [2H] is then key to energy metabolism, driving most fermentation pathways (Janssen, 2010), which has a major impact on ruminant nutrition.

Decreases in methanogenesis have been proposed to increase ruminant performance by allocating [2H] to fermentation pathways more energetically-beneficial to the animals. However, this assumption has not always been confirmed in animal experiments; inhibition of CH_4_ production by up to 30% did not result in greater daily milk production (Haisan et al., 2013; Guyader et al., 2016) or live weight gain (Beauchemin and McGinn, 2005; Brown et al., 2011) in cattle. Consequently, understanding the fate of [2H] spared from decreased CH_4_ production may help to develop CH_4_-mitigating strategies that could simultaneously improve animal performance.

Ungerfeld (2015) suggested that an increase in ruminal [2H] availability following methanogenesis inhibition enhances fermentation pathways that consume [2H], such as formate, valerate and caproate. *In vivo* studies reported an increase in molar percentages of formate (Olijhoek et al., 2016), valerate (Chung et al., 2011), or isovalerate (Martínez-Fernández et al., 2014) when CH_4_ was decreased by 7–29% (expressed as a function of dry matter intake [DMI]). Greater [2H] availability may also stimulate microbial growth or shift biomass composition toward a more reduced fatty acid profile. However, little information has been reported to confirm those relationships and to our knowledge, a simultaneous analysis of the effects of decreasing CH_4_ production on both fermentation end products and microbial biomass production and composition has never been conducted *in vivo*. Using *in vitro* techniques, microbial protein synthesis was either not affected (Romero-Pérez et al., 2015) or was increased (Van Nevel et al., 1969; Guo et al., 2009) with CH_4_ decrease ranging between 66 and 86%, whereas volatile fatty acid (VFA) profiles were differently modified.

Thus, the objective of this experiment was to provide more detailed information on the fate of [2H] when methanogenesis is decreased in the rumen. To this end, various known chemical inhibitors assumed to have different modes of action on CH_4_ production (nitrate, NIT; 3-nitrooxypropanol, NOP; anthraquinone, AQ) were used. In the rumen, NIT decreases methanogenesis by competing for [2H] during dissimilatory nitrate reduction to ammonium (${\text{NH}}_{4}^{+}$; DNRA) or during denitrification leading to nitrous oxide (N_2_O) production (Yang et al., 2016). At the same time, nitrite produced as an intermediary product of DNRA would have a direct toxic effect toward methanogenic *Archaea* (Klüber and Conrad, 1998; Asanuma et al., 2015). Nitrate decreased CH_4_ production in lactating dairy cows (21 g nitrate/kg dry matter [DM], −23.4% CH_4_; Olijhoek et al., 2016), steers (30 g nitrate/kg DM, −29.4% CH_4_; Newbold et al., 2014) and sheep (20 g nitrate/kg DM, −16.5% CH_4_; de Raphélis-Soissan et al., 2014). Nitrooxypropanol is a synthetic compound developed by DSM Nutritional Products Ltd (Kaiseraugst, Switzerland). By positioning itself into the active site of the methyl-coenzyme M reductase, NOP inactivates cofactor F_430_ thereby inhibiting the last step of CH_4_ production in methanogenic *Archaea* (Duin et al., 2016). It decreased methanogenesis in sheep (100 mg/d, −23.7% CH_4_; Martínez-Fernández et al., 2014), lactating dairy cows (2.5 g/d, −6.7% CH_4_; Reynolds et al., 2014) and beef heifers (2.0 g/d, −59.2% CH_4_; Romero-Perez et al., 2015). Anthraquinones are the largest group of quinones (Thomson, 1971) and are naturally present in a large number of plant-derived drugs (Mueller et al., 1999). Their mode of action as methanogenesis inhibitors in the rumen has not been clearly elucidated although they are known to have antibacterial activity (Odom, 1997) by disrupting bacterial membranes (Chan et al., 2011) and bacterial protein synthesis (Anke et al., 1980). In sheep, 9,10-anthraquinone supplementation caused a decrease in CH_4_ production and an accumulation of H_2_, suggesting a direct toxic effect toward methanogenic *Archaea* (500 ppm/d, −50% CH_4_; Kung et al., 2003).

## Materials and methods

### Experimental design and treatments

The experiment was conducted at the Lethbridge Research and Development Centre (Agriculture and Agri-Food Canada). The Rumen Simulation Technique (RUSITEC) was favored over a batch culture or an *in vivo* experiment as it allows for a more precise control of the system while offering the possibility to evaluate the long-term effect of selected methanogenesis inhibitors. Two RUSITEC apparatuses were used, each one consisting of a water bath maintained at 39°C and 8 fermentation vessels of 900 mL working volume automatically and continuously vertically mixed. The experiment was designed as a randomized block with 4 treatments repeated in duplicate in each RUSITEC apparatus (*n* = 4). The treatments were control diet alone (CON; 60% corn silage and 40% cereals and minerals on a DM basis) or supplemented with NIT, NOP or AQ (Table 1). The average organic matter (OM), crude protein (CP), neutral detergent fiber (NDF) and acid detergent fiber (ADF) content of the substrate was 91.1, 21.0, 37.8, and 18.7% of DM. The incubation lasted 19 days. The microbial community was adapted to treatments from day 1 to 7, and measurements started on day 8 until day 19. Supplementation of methanogenesis inhibitors was constant from day 1 to 14 and was then discontinued to study the recovery of fermentation after removal of the methanogenesis inhibitors.

**Table 1:** **Ingredient and chemical composition of the substrates used in the ***in vitro*** rumen simulation technique; treatments were control (CON), nitrate (NIT), 3-nitrooxypropanol (NOP), and anthraquinone (AQ)**.

**Item**	**Substrate**			
	**CON**	**NIT**	**NOP**	**AQ**
**INGREDIENT (% DM)**				
Corn silage	60.0	60.0	60.0	60.0
Barley grain	28.0	28.0	28.0	28.0
Urea	4.26	0	4.26	4.26
Calcium carbonate	4.50	0	4.50	4.50
Calcium ammonium nitrate^a^	0	10.7	0	0
NOP active compound (1,3-propanediol mononitrate)	0	0	0.05	0
NOP carrier (60% SiO_2_, 40% propylene glycol)	0.38	0.38	0.38	0.38
Dicalcium phosphate	2.86	0.92	2.81	2.86
**CHEMICAL COMPOSITION (% DM)**				
OM	89.2	92.3	89.7	93.1
CP	17.9	18.8	23.6	23.6
NPN^b^	2.00	2.00	2.00	2.00
NDF	39.7	35.1	36.5	39.7
ADF	20.3	17.1	17.6	19.8

*DM, dry matter; OM, organic matter; CP, crude protein; NPN, non-protein nitrogen; NDF, neutral detergent fiber; ADF, acid detergent fiber*.

a *5Ca(NO_3_)_2_ NH_4_NO_3_ (75% ${\text{NO}}_{3}^{-}$ in DM)*.

b *Estimated values based on nitrogen content of urea (46.7% of DM) and calcium ammonium nitrate (18.7% of DM)*.

The dose of each additive was selected with an aim of obtaining 80% CH_4_ decrease. Calcium ammonium nitrate was used as the ${\text{NO}}_{3}^{-}$ source (5Ca(NO_3_)_2_·NH_4_NO_3_, 75% ${\text{NO}}_{3}^{-}$ in product DM; Yara International, Oslo, Norway). Given that ${\text{NO}}_{3}^{-}$ had never been tested in RUSITEC conditions, the dose was chosen based on a meta-analysis of *in vivo* experiments (Lee and Beauchemin, 2014) to be equal to 803 mg ${\text{NO}}_{3}^{-}$ vessel^−1^ d^−1^ (8.0% ${\text{NO}}_{3}^{-}$ in substrate DM), equivalent to 1.07 g calcium ammonium nitrate vessel^−1^ d^−1^. Urea and calcium carbonate were added in CON, NOP, and AQ to compensate for the additional non-protein nitrogen (NPN) and calcium provided with calcium ammonium nitrate in NIT.

Dose of NOP (11.6% 1,3-propanediol mononitrate in product DM; DSM Nutritional Products Ltd., Kaiseraugst, Switzerland) was based on the RUSITEC study by Romero-Pérez et al. (2015) and was equal to 5 mg 1,3-propanediol mononitrate vessel^−1^ d^−1^, equivalent to 43.1 mg NOP vessel^−1^ d^−1^. The NOP carrier (a mixture of silica and propylene glycol) was added in equivalent amounts in CON, NIT, and AQ to compensate for any possible effects of the carrier on fermentation.

Dose of 9,10-anthraquinone (97% anthraquinone in product DM; Sigma-Aldrich Corp., Saint Louis, MO) was selected based on the *in vitro* continuous culture incubation by Garcia-Lopez et al. (1996) to be equal to 12.3 mg 9,10-anthraquinone vessel^−1^ d^−1^. Because of poor solubility in water (Garcia-Lopez et al., 1996), AQ was dissolved in 70% (v/v) ethanol (12.3 g/L). One milliliter of this solution was added every day to AQ vessels, as well as 1 mL pure 70% (v/v) ethanol to CON, NIT, and NOP vessels to compensate for the addition of ethanol in AQ vessels.

Corn silage and barley grain were freeze-dried and ground in a Wiley mill (A.H. Thomas, Philadelphia, PA) through a 4-mm screen. Then, substrates were prepared by mixing all ingredients per treatment except for NOP active compound and carrier. Feed samples were then weighed (10 g) into nylon bags (51 μm mesh opening) sealed with heat and stored at room temperature until further use. The active compound and carrier of NOP were kept in the dark and at 4°C as recommended by the supplier, and were individually weighed daily before being added to the vessels during substrate bag exchange.

### Rumen simulation technique

On the first day of the experiment, inoculum was collected from 3 rumen-cannulated cows fed for 1 month with a diet comprised of 65% corn silage, 30% dry rolled barley grain and 5% mineral-vitamin supplement on a DM basis. The maintenance of cannulated animals and the rumen fluid collection procedure were approved by the Institutional Animal Care Committee which operates under the guidelines of the Canadian Council on Animal Care (2009). Rumen samples were taken manually before the morning feeding and strained through 4 layers of cheesecloth. Rumen liquid (~12 L) and solid (~200 g) samples were stored in separate pre-warmed insulated thermos bottles before being used. Artificial saliva was prepared as described by Romero-Pérez et al. (2015) (NaHCO_3_, 9.8 g/L; Na_2_HPO_4_, 3.72 g/L; NaCl, 0.47 g/L; KCl, 0.57 g/L; CaCl_2_.2H_2_O, 0.053 g/L; MgCl_2_.6H_2_O, 0.128 g/L; (NH_4_)_2_SO_4_, 0.3 g/L).

To start the incubation, each fermentation vessel was filled under a CO_2_ stream with 700 mL strained rumen fluid, 200 mL warm artificial saliva, one nylon bag containing 10 g of solid rumen digesta (wet weight) and one nylon bag containing the substrate. After 24 h, the bag containing the solid rumen digesta was replaced with a new substrate bag. Each following day, the oldest bag was replaced with a new one, so that each bag remained in its vessel for 48 h. During bag exchange process, vessels were flushed with pure CO_2_ to maintain anaerobic conditions.

Throughout the experiment, saliva was prepared daily and was continuously infused into each vessel at a rate of 626 mL/d (2.9%/h). The outflows were collected in 1-L volumetric flasks which were connected to 2-L plastic bags for gas collection. Every day, at the time of substrate bag exchange, the gas collection bags were clamped and both flasks and gas collection bags were replaced with new ones.

### Fermentation gases and dissolved end products determination

Every day, at the time of substrate bag exchange, pH was measured in each vessel and volume of gas in collection bags was determined with a gas meter (Alexander-Wright, London) to estimate daily gas production volume. From day 8 to 13 and from day 17 to 19, gas samples (20 mL) were taken in duplicate from gas collection bags for measuring gas composition. Samples were collected with a 26-gauge needle (Becton Dickinson, Franklin Lakes, NJ) and injected into evacuated 6.8-mL Exetainer vials (Labco Ltd, Wycombe, Bucks, UK) for analysis of CH_4_, H_2_, CO_2_, and N_2_O with a gas chromatograph equipped with a thermal conductivity detector (Romero-Pérez et al., 2015). Daily individual gas production volume in each vessel was calculated from daily total gas production and individual gas percentages. Total amount of greenhouse gas (GHG) produced per day, expressed as CO_2_-equivalent (CO_2_-eq), was calculated by multiplying the amount of CH_4_, CO_2_, N_2_O, and H_2_ produced with its 100-year global warming potential (CO_2_ = 1, H_2_ = 5.6, CH_4_ = 28, N_2_O = 265; Derwent et al., 2006; IPCC, 2014).

From day 8 to 11 and from day 15 to 18, dissolved H_2_ (dH_2_) concentrations were measured at several time points (0, 0.5, 1, 1.5, 2, 2.5, 3, 4, 7, 10, 13, 17, and 21 h after substrate bag exchange) with a H_2_ sensor (H_2_-500; Unisense, Aarhus, Denmark) attached to a glass flow-through cell (2 mm internal diameter, 6 mm external diameter). The H_2_-sensor was connected to a microsensor multimeter (Unisense) which in turn was connected to a portable computer running SensorTrace Suite software (Version 2.5.0; Unisense). Each day, 4 vessels (one vessel per treatment) were analyzed such that all 16 vessels were measured in 4 days.

The sensor was polarized (1,000 mV) 8 h before starting the measurement period and was calibrated daily before the first measurement point. For calibration, the flow cell was connected to a closed system using H_2_-impermeable Masterflex Tygon® Chemical tubing (Cole-Parmer Instrument Company, USA), starting and ending in an Erlenmeyer flask maintained in a 39°C-water bath. The flask was filled with tap water which circulated in the system via a peristaltic pump (Model 1001, Medical Technology Products, Inc., Huntington Station, NY). A 2-point calibration curve was created using water without and with H_2_ bubbling (80% H_2_-20% CO_2_ gas mixture). At the time of substrate bag exchange, vessels were fitted with a Masterflex Tygon® Chemical tubing (Cole-Parmer Instrument Company, USA) closed by a 1-way stopcock with Luer connection. At sampling time points, a 40-mL syringe with Luer-lock was connected to the vessel and a fermentation fluid sample (40 mL) was taken and directly injected into the flow cell of the H_2_-sensor. After the measurement, the sample was injected back into the vessel.

From day 8 to 11 and from day 17 to 19, 5-mL samples were taken from the outflow of each vessel for analysis of fermentation end products. Effluent was prevented from further fermentation by adding 4 mL of sodium azide (200 g/L) during sampling days. Samples were mixed with 1 mL of 250 g/L phosphoric acid for analysis of carboxylic acids and alcohols (VFA, formate, lactate, succinate, ethanol and propanol) or 1 mL of 10 mL/L sulfuric acid for ${\text{NH}}_{4}^{+}$ analysis. For nitrate and nitrite analyses, no preservative agent was used. All samples were stored at −20°C until analysis. Concentrations of VFA, lactate, succinate, ethanol and propanol were determined by gas-liquid chromatography (GLC; model 6890; Agilent, Wilmington, DE) with a polar capillary column (30 m × 0.32 mm × 1 μm; ZB-FFAP; Phenomenex Inc., Torrance, CA), a flame ionization detector and helium as carrier gas. For VFA, crotonic acid was used as internal standard with split mode injection. The initial oven temperature was set at 150°C for 1 min before being increased to 195°C for 3 min (5°C/min). The injector and detector temperatures were set at 225°C and 250°C, respectively. For lactate and succinate, malonic acid was employed as internal standard with spotless injections. The injector and detector temperatures were set at 190°C and 250°C, respectively. The oven temperature was maintained at 45°C for 1 min, increased by 30°C/min to 150°C and then by 5°C/min to 190°C, and held at this final temperature for 2 min. Formate concentration was quantified using the GLC method of Richardson et al. (1989) with a Zebron column (30 m × 0.25 mm × 0.5 μm; ZB-1; Phenomenex Inc., Torrance, CA), a flame ionization detector and helium as carrier gas. The oven temperature program started at 50°C for 2 min before increasing to 130°C (5°C/min) and finishing at 240°C (15°C/min) for 4.67 min. The injector and detector temperatures were both set at 275°C. Ammonium concentration was analyzed by the improved Berthelot method (Rhine et al., 1998). Nitrate and nitrite concentrations were determined with water quality test strips (Hach Company, Loveland, CO) on samples taken on days 8, 9, 10, and 11 only, as both components were assumed to be absent from the fermentation liquid during the recovery period.

### Substrate disappearance

One feed sample per treatment was taken before starting the incubation and stored at room temperature for analysis of chemical composition. Dry matter, organic matter (OM), neutral detergent fiber (NDF), and acid detergent fiber (ADF) were analyzed on samples ground in a Wiley mill (A.H. Thomas, Philadelphia, PA) through a 1-mm screen, whereas nitrogen (N) content was determined on ball-ground (mixer mill MM 400; Retsch Inc., Newtown, PA) samples. Dry matter was determined after drying at 135°C for 2 h (method 930.15; AOAC, 2005) and OM after ashing at 550°C for 5 h (method 942.05; AOAC, 2005). Fiber (NDF and ADF) was determined by a sequential procedure (Van Soest et al., 1991) with the ANKOM200 Fiber Analyzer (Ankom Technology Corp., Macedon, NY) after pre-treatment with sodium sulfite and α-amylase and expressed inclusive of residual ash. Nitrogen was analyzed by a combustion method with gas chromatography and thermal conductivity detection (Carlo Erba Instrumentals, Milan, Italy; method 990.03; AOAC, 2005) and crude protein (CP) was calculated as N × 6.25.

Nutrient apparent disappearance (DM, OM, CP) was determined from day 8 to 10 and from day 17 to 19 as described in Romero-Pérez et al. (2015). Briefly, bags collected after 48 h incubation were washed gently under cold running water before being dried at 55°C for 48 h for DM analysis. At the end of the experiment, residues were pooled across days per treatment and chemical composition was analyzed as for feed.

### Microbial protein synthesis

From day 5 to 13, microbes were labeled using ^15^N-enriched (NH_4_)_2_SO_4_ (Sigma Chemical Co., St. Louis, MO; minimum 10 atom% ^15^N) in order to estimate microbial protein synthesis from day 11 to 13. Pellets from microbes associated to the liquid phase and weakly attached to the feed were prepared as described in Ribeiro et al. (2015), before being freeze-dried. Pellets from microbes strongly attached to the feed were prepared from feed residues dried at 55°C for 48 h and ball-ground (Ribeiro et al., 2015). The N content of the 3 microbial fractions was determined as described for feed and the enrichment of ^15^N was subsequently analyzed with a mass spectrometer (NA 1500, Carlo Erba Instruments, Rodano, Italy; Wang et al., 2000).

### Metabolic hydrogen balance calculations

For each treatment, the daily molar production of individual fermentation end products (mmol/d) was calculated. Concentrations of each VFA in each vessel outflow (mM) were multiplied by the daily amount of infused saliva (0.626 L). Volume of CH_4_, H_2_ and N_2_O (mL) was converted to moles using the Ideal Gas Law (1 mol ideal gas = 25.6 L at 39°C and 1.013 × 10^5^ Pa).

Total [2H] produced (mmol/d) was calculated as the sum of [2H] released during daily production of acetate, butyrate and caproate (Table 2). Total [2H] consumed (mmol/d) was calculated as the sum of [2H] used in the production of formate, propionate, valerate, caproate, heptanoate, gaseous H_2_ (gH_2_) and CH_4_ (Table 2). The following assumptions were made: (i) VFA, except branched-chain VFA (isobutyrate, isovalerate, 2-methylbutyrate), were solely produced from glucose fermentation, with fermentation pathways based on Mills et al. (2001) and Ungerfeld and Kohn (2006); (ii) Heptanoate was produced from the condensation of one propionyl-CoA and 2 acetyl-CoA (Bornstein and Barker, 1948); (iii) Branched-chain VFA mainly derived from branched-chain amino acids (valine, leucine, isoleucine) and the associated [2H] balance were not taken into account. As the proportion of ${\text{NO}}_{3}^{-}$ reduced to ${\text{NH}}_{4}^{+}$ and N_2_O was unknown, the total [2H] consumed for NIT treatment was not calculated.

**Table 2:** **Reaction equations used to estimate the production and consumption of metabolic hydrogen ([2H]) during the synthesis of major rumen fermentation end products (presented as underlined in the reaction equations)**.

**Fermentation end product**	**Reaction equation**	**Moles [2H] released/mole end product**
Formate	[2H] + CO_2_ 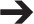 HCOOH	–1
Acetate	C_6_H_12_O_6_ + 2 H_2_O 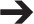 2 CH_3_COOH + 4 [2H] + 2 CO_2_	+2
Propionate	C_6_H_12_O_6_ + 2 [2H] 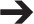 2 CH_3_CH_2_COOH + 2 H_2_O	−1
Butyrate	C_6_H_12_O_6_ 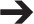 1 CH_3_(CH_2_)_2_COOH + 2 [2H] + 2 CO_2_	+2
Valerate	C_6_H_12_O_6_ + [2H] 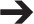 CH_3_(CH_2_)_3_COOH + CO_2_ + 2 H_2_O	−1
Caproate^A^	3 C_6_H_12_O_6_ 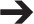 2 CH_3_(CH_2_)_4_COOH + 4 [2H] + 2 H_2_O + 6 CO_2_	+2
Caproate^B^	C_6_H_12_O_6_ + 4 [2H] 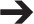 CH_3_(CH_2_)_4_COOH + 4 H_2_O	−4
Heptanoate	2 C_6_H_12_O_6_ + 2 [2H] 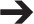 CH_3_(CH_2_)_5_COOH + 2 CO_2_ + CH_3_CH_2_COOH + 4 H_2_O	–2
Methane	CO_2_ + 4 [2H] 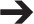 CH_4_ + 2 H_2_O	−4

A *Acetyl-CoA as intermediate*.

B *Propionyl-CoA as intermediate*.

Caproate can be synthesized from the condensation of 2 propionyl-CoA or 2 acetyl-CoA generating 1 butyryl-CoA further condensing with a third acetyl-CoA (Barker et al., 1945; Wallace et al., 2004; Kucek et al., 2016). The first pathway requires the incorporation of 4 moles [2H] per mole of caproate whereas the second one releases 2 moles [2H] per mole of caproate. Given that the proportion of caproate produced by either pathway was unknown, 2 scenarios were assessed to estimate total [2H] produced and consumed. The first scenario assumed that 100% of caproate originated from the condensation of propionyl-CoA (SC1), and the second scenario assumed that 100% of caproate originated from the condensation of 2 acetyl-CoA (SC2).

Finally, for treatments CON, NOP, and AQ, and for each scenario of caproate production, the [2H] balance (mmol/d) was calculated as the difference between [2H] produced (mmol/d) and [2H] consumed (mmol/d) and the percentage of [2H] recovered was calculated as the ratio between [2H] consumed (mmol/d) and [2H] produced (mmol/d).

### Statistics

All statistics were run with SAS software (Version 2.0.4; SAS Institute Inc., Cary, NC). Data were analyzed using the MIXED procedure, with the vessel used as the experimental unit. Treatment and recovery periods were analyzed separately. For dH_2_ concentrations, the model included the fixed effect of treatment. For all other variables, the model included the fixed effects of treatment, day and their interaction, and day was treated as a repeated measure. Variance component was selected as the covariance structure with the lowest Akaike and Bayesian information criteria values for most variables. For all variables, the vessel nested within its treatment was considered as random effect (García-González et al., 2010) and degrees of freedom were adjusted using the Kenward-Roger option. Least-square means are reported throughout the paper and multiple pairwise comparisons between treatments were tested using the PDIFF option. Data were considered significant at *P* < 0.05 and trends were discussed at 0.05 ≤ *P* ≤ 0.10.

Principal component analyses were conducted to illustrate the relationships among variables (*n* = 21; component plots) and treatments (*n* = 4; score plots) using the PRINCOMP procedure. Input data (*n* = 16) consisted of averaged and standardized (STANDARD procedure) data collected during the treatment period. The correlation matrix was prepared with the CORR procedure. Given the large number of computations and to reduce the experimentwise error rate, the raw *P*-values generated from the correlation analyses were subsequently adjusted with the MULTTEST procedure with the false discovery rate (FDR) option. Finally, to compensate for the adjustment of *P*-values, correlations were considered significant at *P* ≤ 0.10.

## Results

### Treatment period

#### Fermentation parameters and nutrient disappearance

Nitrate tended to decrease daily total gas production (27% decrease relative to CON), whereas NOP and AQ had no effect (*P* = 0.083; Table 3). Overall total GHG produced was different among treatments (*P* < 0.001) with NIT and NOP producing less GHG compared to CON and AQ (−73%). Methane percentage was the lowest with NIT and NOP (75% decrease on average relative to CON), whereas AQ decreased CH_4_ by 26% relative to CON (*P* < 0.001). The percentage of gH_2_ was similar between CON and NIT, but was greater with NOP and AQ (5.2- and 3.3-fold increase relative to CON, respectively; *P* < 0.001). Carbon dioxide percentage was similar between CON and AQ, but was greater with NIT and NOP (1.18- and 1.05-fold increase relative to CON, respectively; *P* < 0.001). Nitrous oxide was only detected with NIT (0.04% of total gas; *P* = 0.011). Dissolved H_2_ concentration also differed among treatments, with the greatest concentration recorded for NOP, followed in descending order by CON, NIT and AQ (*P* < 0.001). Inhibiting methanogenesis did not affect DM and OM disappearance of the substrate, but NIT decreased CP disappearance (77.4 and 69.5% for CON and NIT respectively, *P* = 0.028; Table 3).

**Table 3:** **Effects of control (CON), nitrate (NIT), 3-nitrooxypropanol (NOP), and anthraquinone (AQ) on gas production and substrate disappearance using a rumen simulation technique**.

**Item**	**Substrate**				**SEM**	***P*-value^A^**
	**CON**	**NIT**	**NOP**	**AQ**		
**GAS PRODUCTION (L/D)^B^**						
Total	0.74	0.54	0.63	0.76	0.066	0.083
Total GHG (CO_2_-eq)^C^	1.00^a^	0.16^b^	0.34^b^	0.83^a^	0.122	<0.001
**GHG (% OF TOTAL)^B^^,^^D^**						
Methane	17.1^a^	3.6^c^	5.0^c^	12.6^b^	0.84	<0.001
Hydrogen	2.0^c^	1.0^c^	10.3^a^	6.6^b^	0.99	<0.001
Carbon dioxide	80.9^c^	95.4^a^	84.7^b^	80.9^c^	1.19	<0.001
Nitrous oxide	0.00^b^	0.04^a^	0.00^b^	0.00^b^	0.006	0.011
Dissolved hydrogen (μM)^E^	40.8^b^	28.7^c^	53.7^a^	9.7^d^	3.79	<0.001
**SUBSTRATE DISAPPEARANCE (%)^F^**						
DM	47.9	43.3	46.6	45.7	2.07	0.446
OM	47.9	45.2	48.1	46.6	1.97	0.731
CP	77.4^a^	69.5^b^	78.6^a^	75.4^ab^	1.97	0.028

*CO_2_-eq, CO_2_-equivalent; DM, dry matter; GHG, greenhouse gas; OM, organic matter; CP, crude protein*.

A *Within a row, means with different superscripts differ (P < 0.05)*.

B *Average of data collected in all vessels during 6 consecutive days (day 8–13)*.

C *Sum of CH_4_, H_2_, CO_2_, and N_2_O produced corrected for their 100-year global warming potential (CO_2_: 1, CH_4_: 28, N_2_0: 265, H_2_: 5.6; Derwent et al., 2006; IPCC, 2014)*.

D *Gas percentages are based on the sum of CH_4_, H_2_, CO_2_, and N_2_O produced*.

E *Average of data collected in 4 vessels/day during 4 consecutive days (day 8–11)*.

F *Average of data collected in all vessels during 3 consecutive days (day 8–10)*.

Medium pH was similar among treatments and averaged 6.95 (Table 4). Total VFA concentration tended to differ among treatments (*P* = 0.098), with the lowest concentration for NIT (22.3% decrease relative to CON). Lactate and succinate were not detected for any treatment. Treatments did not modify formate, but tended to change propionate molar percentage (*P* = 0.061), numerically decreasing it with NIT and increasing it with NOP and AQ. Compared to CON, NIT increased acetate molar percentage (+16.0 percentage units) and decreased butyrate, isobutyrate, valerate, isovalerate, caproate and heptanoate molar percentages (−1.8, −0.26, −1.51, −6.77, −3.29, and −1.09 percentage units, respectively; *P* < 0.001). In contrast, NOP and AQ both decreased acetate molar percentage (−7.4 and −5.6 percentage units, respectively) and increased caproate (+2.24 and +4.78 percentage units, respectively) and heptanoate molar percentages (+0.74 and +1.58 percentage units, respectively) (*P* < 0.001). Nitrooxypropanol also increased butyrate and valerate (+2.10 and +2.70 percentage units, respectively), but decreased isovalerate (−1.58 percentage units) molar percentages (*P* < 0.001). Anthraquinone decreased isobutyrate (−0.09 percentage units; *P* < 0.001) without affecting butyrate, valerate and isovalerate molar percentages. Only NIT modified acetate:propionate (+2.2 ratio points relative to CON; *P* < 0.001) and (acetate+butyrate):propionate (+2.19 ratio points relative to CON; *P* = 0.002).

**Table 4:** **Effects of control (CON), nitrate (NIT), 3-nitrooxypropanol (NOP), and anthraquinone (AQ) on fermentation variables using a rumen simulation technique**.

**Item^B^**	**Substrate**				**SEM**	***P*-value^A^**
	**CON**	**NIT**	**NOP**	**AQ**		
pH	6.92	6.95	6.97	6.96	0.016	0.200
Total VFA (mM)	65.5	50.9	59.7	63.8	4.02	0.098
**VFA PROFILE (mol/100 mol)**						
Formate	5.76	5.52	7.21	5.68	0.664	0.293
Acetate (A)	52.1^b^	68.1^a^	44.7^c^	46.5^c^	0.82	<0.001
Propionate (P)	12.6	11.3	13.8	11.3	0.69	0.061
Butyrate (B)	15.3^b^	13.5^c^	17.4^a^	16.3^a^^b^	0.47	<0.001
Isobutyrate	0.56^a^	0.30^c^	0.53^a^^b^	0.47^b^	0.022	<0.001
Valerate	5.56^b^	4.05^c^	8.26^a^	5.92^b^	0.187	<0.001
Isovalerate^C^	7.61^a^	0.84^c^	6.03^b^	6.83^a^^b^	0.268	<0.001
Caproate	4.72^c^	1.43^d^	6.96^b^	9.50^a^	0.671	<0.001
Heptanoate	1.57^c^	0.48^d^	2.31^b^	3.15^a^	0.222	<0.001
A/P (mol/mol)	4.16^b^	6.36^a^	3.28^b^	4.18^b^	0.391	<0.001
(A+B)/P (mol/mol)	5.38^b^	7.57^a^	4.54^b^	5.64^b^	0.431	0.002
Ethanol (mM)	5.89^bc^	9.92^a^	7.68^b^	4.25^c^	0.600	<0.001
n-propanol (mM)	0.19^b^	0.16^b^	0.41^a^	0.16^b^	0.044	0.004
Ammonium (mM)	14.1^b^	11.3^c^	19.2^a^	19.6^a^	0.54	<0.001
Nitrate (mM)	0.00^b^	0.27^a^	0.00^b^	0.00^b^	0.053	0.001
Nitrite (mM)	0.00^b^	0.03^a^	0.00^b^	0.00^b^	0.008	0.036

*VFA, volatile fatty acid*.

A *Within a row, means with different superscripts differ (P < 0.05)*.

B *Average of data collected in all vessels during 3 consecutive days (day 17–19)*.

C *Co-elutes with 2-methylbutyrate*.

Ethanol concentration increased with NIT (1.68-fold increase relative to CON; *P* < 0.001) but was not affected by NOP and AQ. No treatment other than NOP affected n-propanol concentration (2.16-fold increase relative to CON; *P* = 0.004). Ammonium concentration was greater for NOP and AQ (1.38-fold increase relative to CON on average) and lower for NIT (19.9% decrease relative to CON) (*P* < 0.001). Finally, nitrate and nitrite were detected in outflow from NIT, showing that a maximum of 94% of the daily amount of ${\text{NO}}_{3}^{-}$ supplied could have been fully reduced to ${\text{NH}}_{4}^{+}$.

Microbial N production was modified differently depending upon the treatment (Table 5; *P* < 0.05). Treatment NIT increased both total microbial N production and efficiency of microbial protein synthesis (1.17- and 1.16-fold increase relative to CON, respectively), whereas AQ decreased both variables (13.3 and 17.3% decrease relative to CON, respectively). Treatment NOP had no effect on either variable.

**Table 5:** **Effects of control (CON), nitrate (NIT), 3-nitrooxypropanol (NOP), and anthraquinone (AQ) on microbial protein synthesis using a rumen simulation technique**.

**Item^B^**	**Substrate**				**SEM**	***P*-value^A^**
	**CON**	**NIT**	**NOP**	**AQ**		
**MICROBIAL NITROGEN PRODUCTION (mg/d)**						
Total	59.8^b^	69.7^a^	54.2^bc^	51.7^c^	2.68	0.002
Microbes strongly attached to feed	33.6	36.6	33.8	30.4	1.64	0.084
Microbes weakly attached to feed	5.6	4.8	4.9	4.4	0.58	0.554
Microbes associated to liquid phase	22.4^bc^	28.8^ab^	15.6^c^	16.9^c^	2.42	0.008
E_MPS_	15.9^b^	18.5^a^	14.1^bc^	13.3^c^	0.91	0.001

*E_MPS_, efficiency of microbial protein synthesis (grams microbial nitrogen produced per kilogram organic matter fermented)*.

A *Within a row, means with different superscripts differ (P < 0.05)*.

B *Average of data collected in all vessels during 3 consecutive days (day 11–13)*.

#### Variables and treatments clustering by principal component analysis

**Figure 1:**
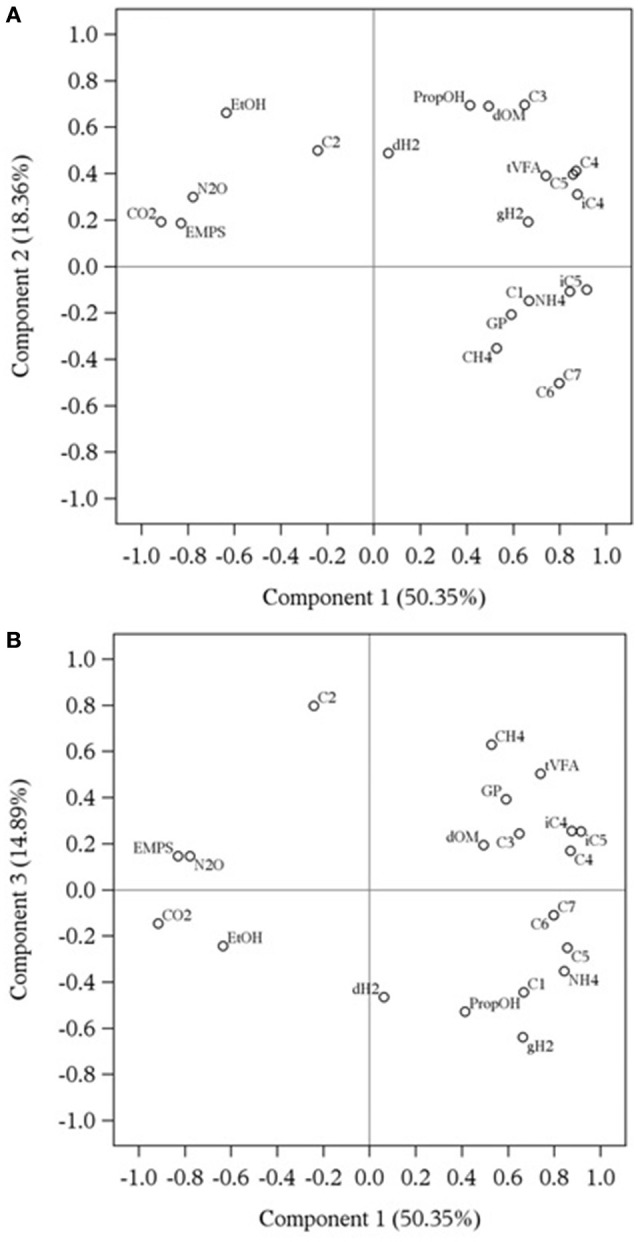
**Component pattern plots obtained by principal component analysis describing the relationship among rumen fermentation variables affected by methanogenesis inhibitors using a rumen simulation technique, along components 1 and 2 (A)** and components 1 and 3 **(B)**. dH_2_: dissolved hydrogen (μM); GP, total gas production (mL/d); CH_4_, methane (%); gH_2_, gaseous hydrogen (%); CO_2_, carbon dioxide (%); N_2_O, nitrous oxide (%); dOM, organic matter disappearance (%); E_MPS_, efficiency of microbial protein synthesis (g microbial N/kg organic matter fermented); tVFA, total volatile fatty acid concentration (mM); C1, formate (mM); C2, acetate (mM); C3, propionate (mM); iC4, isobutyrate (mM); C4, butyrate (mM); C5, valerate (mM); iC5, isovalerate (mM); C6, caproate (mM); C7, heptanoate (mM); EtOH, ethanol (mM); NH_4_, ammonium (mM); PropOH, propanol (mM). Gas percentages are based on the sum of CH_4_, H_2_, CO_2_, and N_2_O produced.

Representation of response variables along components 1 and 2 and along components 1 and 3 is presented in Figures 1A,B. The first, second and third principal components explained 83.6% of the total variation of the dataset. The component and score plots between components 2 and 3 are not presented as they did not provide additional information. In Figure 1, close variables mean that they are positively correlated whereas variables on opposite directions of the same axis are negatively correlated. Two variables situated on orthogonal axes are not correlated. The correlation matrix between fermentation variables is presented in Supplementary Table 1.

Based on Eigenvalues with absolute value greater than 0.20 (Table 6), CO_2_ and N_2_O percentages, concentrations of ${\text{NH}}_{4}^{+}$, butyrate, valerate and branched-chain VFA as well as efficiency of microbial protein synthesis mostly contributed to the first component. Carbon dioxide was positively correlated with nitrous oxide (*r* = 0.83, *P* < 0.001) and efficiency of microbial protein synthesis (*r* = 0.71, *P* = 0.011), although negatively correlated with butyrate (*r* = −0.72, *P* = 0.010), isobutyrate (*r* = −0.83, *P* < 0.001), valerate (*r* = −0.65, *P* = 0.026), isovalerate (*r* = −0.94, *P* < 0.001) and ${\text{NH}}_{4}^{+}$ (*r* = −0.66, *P* = 0.020). Nitrous oxide was also positively correlated with efficiency of microbial protein synthesis (*r* = 0.80, *P* = 0.002) and negatively correlated with isobutyrate (*r* = −0.59, *P* = 0.050), valerate (*r* = −0.50, *P* = 0.100), isovalerate (*r* = −0.79, *P* = 0.003) and ${\text{NH}}_{4}^{+}$ (*r* = −0.66, *P* = 0.023). Efficiency of microbial protein synthesis was negatively correlated with butyrate (*r* = −0.63, *P* = 0.029), isobutyrate (*r* = −0.55, *P* = 0.0681), valerate (*r* = −0.64, *P* = 0.029), isovalerate (*r* = −0.70, *P* = 0.013) and ${\text{NH}}_{4}^{+}$ (*r* = −0.84, *P* < 0.001). The four VFA were positively correlated together (*r* > 0.67, *P* < 0.050) and with ${\text{NH}}_{4}^{+}$ (*r* > 0.55, *P* < 0.068).

**Table 6:** **Eigenvalues obtained by principal component analysis describing the contribution to components 1, 2, and 3 of rumen fermentation variables affected by methanogenesis inhibitors using a rumen simulation technique**.

**Item**	**Component 1 (50.35%)**	**Component 2 (18.36%)**	**Component 3 (14.89%)**
Dissolved hydrogen (μM)	0.019	0.249	−0.263
Total gas production (mL/d)	0.182	−0.105	0.222
Methane (%)^A^	0.162	−0.180	0.356
Gaseous hydrogen (%)^A^	0.204	0.098	−0.361
Carbon dioxide (%)^A^	−0.282	0.098	−0.083
Nitrous oxide (%)^A^	−0.239	0.152	0.083
OM disappearance (%)	0.152	0.352	0.110
E_MPS_ (g microbial N/kg OM fermented)	−0.255	0.095	0.083
Total VFA (mM)	0.228	0.199	0.284
Acetate (mM)	−0.074	0.254	0.451
Propionate (mM)	0.200	0.354	0.138
Isobutyrate (mM)	0.270	0.158	0.145
Butyrate (mM)	0.268	0.209	0.096
Valerate (mM)	0.264	0.202	−0.142
Isovalerate (mM)	0.281	−0.051	0.143
Caproate (mM)	0.245	−0.256	−0.062
Heptanoate (mM)	0.245	−0.256	−0.062
Ethanol (mM)	−0.195	0.337	−0.138
Ammonium (mM)	0.259	−0.055	−0.199
Formate (mM)	0.205	−0.075	−0.251
n-propanol (mM)	0.127	0.353	−0.298

*OM, organic matter; E_MPS_, efficiency of microbial protein synthesis; N, nitrogen; VFA, volatile fatty acid*.

A *Gas percentages are based on the sum of CH_4_, H_2_, CO_2_, and N_2_O produced*.

Propionate, caproate, heptanoate, ethanol, and propanol concentrations and OM disappearance were the main contributors of the second component (Table 6). Propionate was only positively correlated with propanol (*r* = 0.62, *P* = 0.038) and OM disappearance (*r* = 0.80, *P* = 0.002). Propanol and OM disappearance were positively correlated (*r* = 0.55, *P* = 0.068) as were caproate and heptanoate (*r* = 1.00, *P* < 0.001). Ethanol was negatively correlated with caproate and heptanoate (*r* = −0.86, *P* < 0.001).

Total gas production, CH_4_ and H_2_ percentages as well as concentrations of dH_2_, total VFA, formate and acetate contributed to the third component (Table 6). Dissolved H_2_ concentration was not correlated with any variable. A positive correlation was observed between total gas production and CH_4_ percentage (*r* = 0.51, *P* = 0.094), total gas production and total VFA concentration (*r* = 0.50, *P* = 0.100) and CH_4_ percentage and total VFA concentration (*r* = 0.52, *P* = 0.089). Finally, formate was positively correlated with gH_2_ percentage (*r* = 0.60, *P* = 0.046) and negatively correlated with acetate (*r* = −0.58, *P* = 0.057), whereas acetate and gH_2_ were negatively correlated (*r* = −0.53, *P* = 0.078).

**Figure 2:**
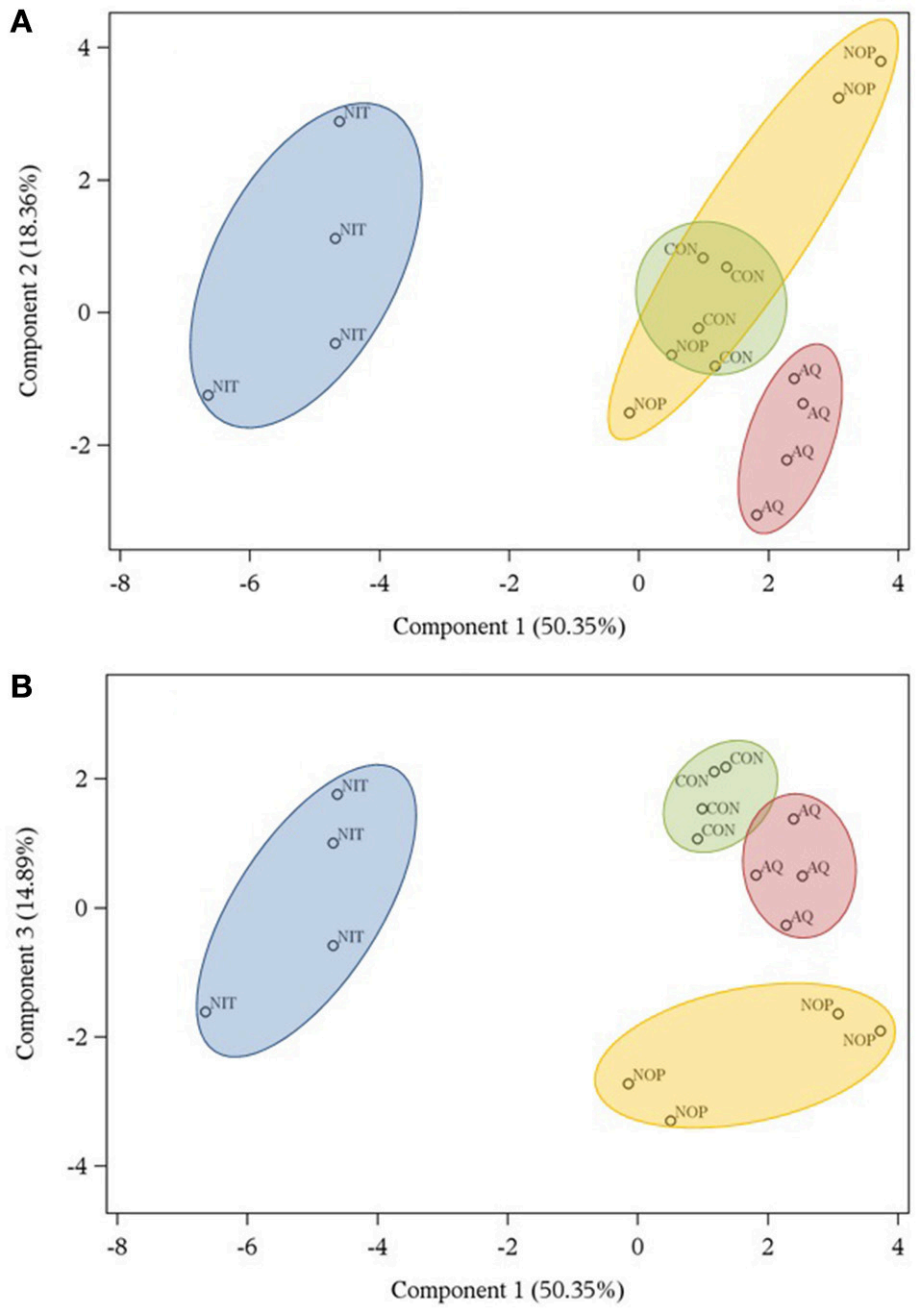
**Score plots obtained by principal component analysis describing the distribution of treatments (control, CON; nitrate, NIT; 3-nitrooxypropanol, NOP; anthraquinone, AQ) tested with a rumen simulation technique along components 1 and 2 (A)** and components 1 and 3 **(B)**.

Representation of treatments along components 1 and 2 and along components 1 and 3 is shown in Figures 2A,B. Treatment NIT created a distinct group along component 1 because of greater efficiency of microbial protein synthesis and N_2_O production and also because of lower gH_2_ production and branched-chain VFA. Treatment AQ was separated from other treatments in component 2 as it was characterized by high concentrations of caproate and heptanoate and low acetate concentration. Finally, NOP clustered along component 3 mostly because of high dH_2_ and propanol concentrations, but also because of numerically higher formate concentration.

#### Metabolic hydrogen balance

Total [2H] production was not different among treatments, regardless of caproate metabolic pathway used (Table 7). However, NIT decreased the amount of [2H] produced from butyrate (30.4% decrease relative to CON; *P* = 0.036), and NOP tended to decrease [2H] production from acetate (21.8% decrease relative to CON; *P* = 0.053). In SC2, AQ and NIT respectively produced the greatest and the lowest amount of [2H] from caproate (*P* < 0.001) compared to the other treatments.

**Table 7:** **Effects of control (CON), nitrate (NIT), 3-nitrooxypropanol (NOP), and anthraquinone (AQ) on metabolic hydrogen ([2H]) balance using a rumen simulation technique**.

**Item^B^**	**Substrate**				**SEM**	***P*-value^A^**
	**CON**	**NIT**	**NOP**	**AQ**		
**[2H] PRODUCTION (mmol/d)**						
Acetate	42.7	43.1	33.4	37.2	2.53	0.053
Butyrate	12.5^a^	8.7^b^	13.1^a^	13.1^a^	1.08	0.036
Caproate (SC1)	0	0	0	0	0	–
Caproate (SC2)	3.88^c^	0.92^d^	5.01^b^	7.51^a^	0.302	<0.001
Total (SC1)	55.2	51.8	46.5	50.3	3.54	0.410
Total (SC2)	59.1	52.7	51.5	57.9	3.41	0.351
**[2H] CONSUMPTION (mmol/d)**						
Formate	2.33^a^	1.65^b^	2.58^a^	2.25^a^	0.157	0.001
Propionate	5.17	3.71	5.25	4.53	0.549	0.219
Valerate	2.28^b^	1.33^c^	3.09^a^	2.35^b^	0.193	<0.001
Caproate (SC1)	7.75^c^	1.83^d^	10.02^b^	15.02^a^	0.605	<0.001
Caproate (SC2)	0	0	0	0	0	–
Heptanoate	1.29^c^	0.30^d^	1.66^b^	2.49^a^	0.100	<0.001
Methane	4.54^a^	0.24^b^	1.00^b^	3.79^a^	0.562	<0.001
Hydrogen	0.16^b^	0.03^b^	0.46^a^	0.65^a^	0.096	<0.001
Total (SC1)	23.5^b^	–	24.0^b^	30.8^a^	1.08	<0.001
Total (SC2)	15.8	–	13.7	16.0	0.89	0.153
**[2H] BALANCE^C^**						
mmol/d (SC1)	31.7^a^	–	21.4^b^	19.9^b^	2.50	0.016
mmol/d (SC2)	43.3^a^	–	36.2^b^	42.1^a^	1.71	0.034
% (SC1)	42.4^b^	–	54.3^a^	61.2^a^	2.99	0.004
% (SC2)	26.6	–	27.7	27.5	1.14	0.788

A *Within a row, means with different superscripts differ (P < 0.05)*.

B *SC1: 100% of caproate is produced from propionyl-CoA; SC2: 100% of caproate is produced from acetyl-CoA*.

C *mmol/d = [2H] produced—[2H] consumed; % = [2H] consumed/[2H] produced*.

Treatments modified all pathways of [2H] consumption except propionate (Table 7). Nitrate decreased [2H] uptake into formate and valerate (29.2 and 41.7% decrease relative to CON, respectively; *P* = 0.001) whereas NOP increased [2H] diverted toward valerate (1.36-fold increase relative to CON). In SC1, [2H] uptake into caproate was greater with AQ, followed by NOP, CON, and NIT (*P* < 0.001). Both NOP and AQ increased [2H] directed toward heptanoate (0.37- and 1.93-fold increase relative to CON, respectively) whereas NIT decreased [2H] consumption in heptanoate formation (76.7% decrease relative to CON; *P* < 0.001). The amount of [2H] used for methanogenesis was lower for NIT and NOP (86% decrease on average relative to CON; *P* < 0.001) and was not modified with AQ. Nitrate did not modify the amount of [2H] incorporated into gH_2_, but NOP and AQ increased it (3.47-fold increase on average relative to CON; *P* < 0.001). Finally, in SC1, NOP presented similar rates of total [2H] consumption as did CON whereas AQ increased total [2H] uptake (1.31-fold increase relative to CON; *P* < 0.001). In SC2, total [2H] consumption was similar among CON, NOP and AQ.

Overall, treatments modified [2H] balance differently (*P* < 0.001; Table 7). Expressed as daily millimoles, the [2H] balance in SC1 was similar between NOP and AQ but lower than CON (34.9% decrease on average relative to CON; *P* = 0.016). In SC2, the [2H] balance of NOP was also lower than CON (16.4% decrease relative to CON; *P* = 0.034). Total [2H] consumed expressed as a proportion of total [2H] produced was similar between CON, NOP and AQ in SC2, but was greater for NOP and AQ in SC1 (1.36-fold increase on average relative to CON; *P* = 0.004).

### Recovery period

**Figure 3:**
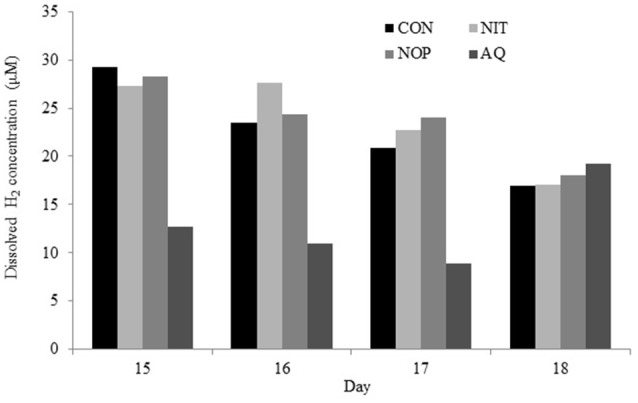
**Effects of control (CON), nitrate (NIT), 3-nitrooxypropanol (NOP), and anthraquinone (AQ) on dissolved H_**2**_ concentration after 1 (day 15), 2 (day 16), 3 (day 17), and 4 (day 18) days of treatment withdrawal (recovery period; day 14–19) using a rumen simulation technique**.

Within treatments, data comparison between measurement and recovery periods presented some differences, most probably because of the microbial evolution of the system throughout time and discontinuation of the treatments. Therefore, data between the two experimental periods were not compared, but comparisons among treatments within each period can still be considered. After removal of methanogenesis inhibitors, total gas production and composition (day 17–19) were similar among treatments, except for gH_2_ percentage which remained higher with AQ (2.74-fold increase relative to CON, *P* = 0.028; Table 8). Dissolved H_2_ still differed among previous treatments (*P* = 0.019) with a lower concentration for AQ (43% decrease relative to CON). However, dH_2_ concentrations for AQ recovered to values similar to those of CON on the last day of measurement (day 18; Figure 3). Substrate degradation was only affected by NOP, with lower DM (*P* = 0.025) and OM (*P* = 0.018) disappearance and a tendency (*P* = 0.104) for lower CP disappearance (6.3, 6.7, and 4.0 percentage units decrease relative to CON, respectively).

**Table 8:** **Effects of control (CON), nitrate (NIT), 3-nitrooxypropanol (NOP), and anthraquinone (AQ) on gas production and substrate disappearance after treatment withdrawal (recovery period; day 14–19) using a rumen simulation technique**.

**Item**	**Substrate**				**SEM**	***P*-value^A^**
	**CON**	**NIT**	**NOP**	**AQ**		
Total gas (L/d)^B^	0.80	1.01	0.92	0.76	0.115	0.399
**GAS (% of TOTAL)^B^**						
Methane	18.6	16.3	18.1	16.2	1.13	0.336
Hydrogen	1.36^b^	2.01^b^	1.31^b^	3.72^a^	0.567	0.028
Carbon dioxide	80.0	81.7	80.7	80.1	1.11	0.668
Nitrous oxide	0.00	0.00	0.00	0.00	0.003	0.325
Dissolved hydrogen (μM)^C^	22.7^a^	23.7^a^	23.7^a^	12.9^b^	2.35	0.019
**SUBSTRATE DISAPPEARANCE (%)^B^**						
DM	50.0^a^	49.4^a^	43.7^b^	48.8^a^	1.54	0.025
OM	59.5^a^	60.6^a^	52.8^b^	58.5^a^	1.55	0.018
CP	74.5	76.1	70.5	73.0	1.48	0.104

*DM, dry matter; OM, organic matter; CP, crude protein*.

A *Within a row, means with different superscripts differ (P < 0.05)*.

B *Average of data collected in all vessels during 3 consecutive days (day 17–19); gas percentages are based on the sum of CH_4_, H_2_, CO_2_ and N_2_O produced*.

C *Average of data collected in 4 vessels/day during 4 consecutive days (day 15–18)*.

Total VFA concentration and pH were similar among previous treatments but individual VFA molar percentages were still different during recovery (Table 9). Compared to CON, NOP and AQ decreased acetate molar percentage (−3.7 and −3.8 percentage units, respectively; *P* = 0.001), AQ decreased propionate molar percentage (−1.3 percentage units; *P* = 0.039) and NIT increased butyrate molar percentage (+2.7 percentage units; *P* = 0.023). Except for isobutyrate, minor VFA molar percentages were also different among treatments. Compared to CON, valerate molar percentage was greater with NIT and NOP (+1.35 and +1.56 percentage units, respectively; *P* = 0.005), isovalerate molar percentage was lower with NIT (−5.85 percentage units; *P* < 0.001) and caproate and heptanoate molar percentages were greater with AQ (+2.78 and +0.92 percentage units, respectively; *P* < 0.001). Volatile fatty acid ratios were not modified by treatments (4.01 and 5.18 on average for acetate:propionate and (acetate+butyrate):propionate ratio), as well as ethanol and n-propanol concentrations. Finally, ${\text{NH}}_{4}^{+}$ concentrations were similar among NIT, NOP, and AQ, but lower for all treatments compared to CON (18% decrease on average; *P* = 0.040).

**Table 9:** **Effects of control (CON), nitrate (NIT), 3-nitrooxypropanol (NOP), and anthraquinone (AQ) on fermentation variables after treatment withdrawal (recovery period; day 14–19) using a rumen simulation technique**.

**Item^B^**	**Substrate**				**SEM**	***P*-value^A^**
	**CON**	**NIT**	**NOP**	**AQ**		
pH	6.96	6.95	6.94	6.93	0.018	0.641
Total VFA (mM)	54.6	60.0	52.2	59.4	3.28	0.311
**VFA PROFILE (mol/100 mol)**						
Acetate (A)	52.0^a^	54.0^a^	48.3^b^	48.2^b^	0.88	0.001
Propionate (P)	13.2^a^	14.0^a^	12.9^a^^b^	11.9^b^	0.50	0.039
Butyrate (B)	13.6^b^	16.3^a^	15.1^a^^b^	14.3^b^	0.57	0.023
Isobutyrate	0.43	0.37	0.40	0.44	0.020	0.143
Valerate	5.61^b^	6.96^a^	7.17^a^	5.25^b^	0.362	0.005
Isovalerate^C^	6.75^a^	0.90^b^	6.76^a^	7.71^a^	0.398	<0.001
Caproate	6.39^bc^	5.57^c^	7.00^b^	9.17^a^	0.405	<0.001
Heptanoate	2.12^bc^	1.85^c^	2.32^b^	3.04^a^	0.134	<0.001
A/P	4.00	4.11	3.81	4.13	0.174	0.557
(A+B)/P	5.04	5.31	5.02	5.34	0.219	0.579
Ethanol (mM)	5.65	5.49	4.43	4.71	0.772	0.624
n-propanol (mM)	0.05	0.12	0.02	0.05	0.028	0.127
Ammonium (mM)	27.0^a^	22.0^b^	21.5^b^	23.2^b^	1.32	0.040

*VFA, volatile fatty acid*.

A *Within a row, means with different superscripts differ (P < 0.05)*.

B *Average of data collected in all vessels during 3 consecutive days (day 17–19)*.

C *Co-elutes with 2-methylbutyrate*.

## Discussion

Although the high levels of CH_4_ production inhibition obtained in this study are rarely achieved *in vivo* mainly due to other undesirable issues related to intake of high doses of tested CH_4_ inhibitors, the present approach was useful for understanding redirection of [2H] due to a decrease in methanogenesis. Even though the goal of this study was not to make a direct comparison of the CH_4_ efficiency of additives, the choice of treatments provided an interesting contrast, as each methanogenesis inhibitor had a different mode of action on rumen fermentation, as shown by the principal component analyses.

### Specific effects of methanogenesis inhibitors on metabolic hydrogen fate

#### Reduction of metabolic hydrogen availability with nitrate

Nitrate decreased CH_4_ production (mL) by 91%, corresponding to an 11.4% CH_4_ decrease per 1% nitrate daily added, assuming a full reduction to ${\text{NH}}_{4}^{+}$. This efficiency is in the range of previous *in vivo* experiments (12.2%, Van Zijderveld et al., 2010; 12.5%, Lund et al., 2014; 7.3%, Lee et al., 2015; 9.2 and 6.8%, Veneman et al., 2015). Nitrous oxide was detected with nitrate, thereby supporting the occurrence of denitrification (Petersen et al., 2015; Latham et al., 2016). However, it can be estimated that only 0.02% of the total amount of nitrate added daily was converted to N_2_O, indicating that DNRA must be the major nitrate degradation pathway. As a consequence of this small increase of N_2_O, the GHG mitigation efficiency of NIT was only slightly affected.

The numerical decrease in gH_2_ production and dH_2_ concentration with NIT supports the assumed mode of action of nitrate acting as a [2H] sink. However, this result contradicts previous batch culture study reporting a numerical increase of gaseous H_2_ for nitrate concentrations higher than 4 mM (Guyader et al., 2015b), as well as *in vivo* experiments showing an increase of gaseous (Lund et al., 2014; Veneman et al., 2015) and dissolved (Guyader et al., 2015a) H_2_ when nitrate was fed to cows up to 23 g/kg DM. Discrepancy among studies may be related with dosage, which was four times greater in the present study in order to inhibit CH_4_ production by at least 80%. One may assume that as the dose of nitrate increases, direct toxicity of nitrite on methanogens gains importance as a mechanism of inhibition of methanogenesis over competition for reducing equivalents. A dose response study to examine effects of nitrate on dH_2_ concentration would provide insights into the effect of nitrate on [2H] production and incorporation.

Nitrate increased acetate molar percentage but decreased all other VFA except propionate, which was not modified by any of the treatments. Several studies also reported an increase of acetate molar percentage with nitrate supplementation (El-Zaiat et al., 2014; Guyader et al., 2015a; Veneman et al., 2015). A shift toward acetate, whose formation from carbohydrates results in the release of [2H], could have been caused by lower [2H] availability as shown by the lower gH_2_ and dH_2_ when NIT was used. In agreement, a negative correlation was observed between gH_2_ and acetate supporting the negative relationship reported by Wang et al. (2016) between dH_2_ concentration and molar percentage of acetate.

The decrease in isobutyrate, isovalerate and 2-methylbutyrate with NIT strongly suggests a decrease in deamination of branched-chain amino acids. Zhou et al. (2011), Zhou et al. (2012), and Patra and Yu (2013) also observed a decrease in isovalerate and isobutyrate with CH_4_ inhibition up to 70% with nitrate addition in batch cultures. Hino and Russell (1985) reported a negative effect of [2H] availability in reduced cofactors and dyes on fermentation of highly reduced amino acids. Because NIT shifted fermentation toward acetate (i.e., a [2H]-releasing pathway), the decrease in deamination of branched-chain amino acids by nitrate may perhaps have been caused by nitrite toxicity on amino acids fermenting organisms, rather than changes in the flow of metabolic hydrogen. A decrease of reduced amino acids concentrations may impact the overall ruminal fermentation of amino acids and increase the proportion of undegradable proteins flowing out of the rumen. This possible decrease in amino acids degradation may be one factor explaining the lower CP degradation and ${\text{NH}}_{4}^{+}$ concentration with NIT, which may also be explained by the partial ${\text{NO}}_{3}^{-}$ reduction and, to a lower extent, the greater daily microbial protein production. Guo et al. (2009) also found lower ${\text{NH}}_{4}^{+}$ concentration and higher microbial N concentration in batch cultures supplemented with nitrate, confirming the potential beneficial effect of nitrate on microbial protein synthesis.

Finally, ethanol concentrations increased with NIT supporting the hypothesis that this alcohol may have a role in disposing reducing equivalents spared from methanogenesis (Ungerfeld et al., 2003). Overall, NIT stimulated [2H] producing pathways as shown by the greater acetate molar percentage. Based on the amount of nitrate added daily, the amount of nitrate and nitrite found in the effluent and the production of N_2_O, the amount of [2H] diverted toward DNRA and denitrification was estimated to be equal to 51.6 mmol/d assuming that 4 moles [2H] are used within each reaction pathway. Then, [2H] recovery in SC1 and SC2 would be equal to 123.5 and 117.9%, respectively (data not shown). This calculation may be biased due to the use of sodium azide to stop the fermentations in the outflow. Indeed, sodium azide may react with nitrate or nitrite to form N_2_O (McIlvin and Altabet, 2005), leading to a potential overestimation of the denitrification pathway. The estimated [2H] balance greater than 100% shows that either some [2H] producing pathways have not been taken into account and/or that the amount of [2H] directed toward DNRA has been overestimated. The use of labeled nitrate to track its decomposition into nitrite, ${\text{NH}}_{4}^{+}$ and N_2_O may help understanding its degradation.

#### Increasing amount of reduced products with nitrooxypropanol

Nitrooxypropanol is a hydrosoluble compound quickly degradable (Duin et al., 2016). As a consequence, its effects on rumen fermentation probably take place in a limited period of time. However, this property did not prevent NOP from decreasing methanogenesis by 80%, which agrees with similar CH_4_-mitigating efficiency observed in a previous RUSITEC study by Romero-Pérez et al. (2015) (76% CH_4_ reduction with 5 mg 1,3-propanediol mononitrate vessel^−1^ d^−1^). The lower CH_4_ production with NOP was associated with an increase of gH_2_ and dH_2_, showing that [2H] spared from decreased methanogenesis was not fully re-allocated toward other fermentation pathways. However, given that NOP decreased CH_4_ production by 1.05 mmoles and that 1 mole CH_4_ is produced from 4 moles [2H], availability of [2H] increased by 4.21 mmoles. As NOP increased gH_2_ by 0.38 mmoles, it means that 9% of reducing equivalents not used in CH_4_ formation ended up in accumulated H_2_. This percentage is close to a previous meta-analysis estimate (5.8%) based on 100% methanogenesis inhibition in continuous culture (Ungerfeld, 2015).

Another part of [2H] spared from methanogenesis was re-directed toward the production of VFA that requires a net incorporation of [2H] when produced from glucose (valerate, heptanoate, and caproate produced via propionyl-CoA) or VFA whose production results in less release of [2H] per unit of glucose compared to acetate (butyrate and caproate via acetyl-CoA/butyryl-CoA). In agreement with Ungerfeld (2015), [2H] diverted toward propionate was not modified by a decrease in methanogenesis.

Propanol concentration increased during the treatment period, most probably as a result of NOP degradation. Indeed, one mole of NOP is degraded to one mole of propanol through propanediol and propanal (Duin et al., 2016). The calculated moles of additional propanol with NOP (0.14 mmoles/d increase relative to CON) largely exceeded the daily molar supplementation of NOP (0.04 mmoles/d). Assuming that the full amount of NOP was degraded to propanol and that the dilution rate of propanol was similar to the aqueous phase, calculations showed that the greater propanol concentration was likely related to its accumulation over days (data not shown).

No additional [2H] was directed toward microbial protein synthesis, as microbial efficiency was similar to that of CON, confirming the results of Romero-Pérez et al. (2015). Overall, the [2H] balance was similar between CON and NOP. Depending on the caproate production scenario assumed, between 46 and 72% of [2H] produced was still not accounted for in the balance, indicating that several [2H] sinks were missed in the current experiment.

Upon removal of the NOP treatment, gas production and composition, as well as propanol concentration, recovered to control levels even though acetate molar percentage did not fully recover and remained lower than CON. Similarly, valerate molar percentage was still greater than CON during recovery. These few remaining differences between CON and NOP may indicate changes of microbiota during the NOP treatment period, that require more than 5 days to fully recover.

#### Increasing amount of metabolic hydrogen wastage with anthraquinone

Anthraquinone decreased CH_4_ production by only 27%, which was substantially less than expected based on previous experiments. Indeed, in a continuous culture, a lower dosage of anthraquinone decreased methanogenesis by 62% (Garcia-Lopez et al., 1996). Even though reasons for the lower effect observed in the present work are not clear, the decrease in methanogenesis was sufficient to observe several modifications in the fermentation profile.

Anthraquinone increased gH_2_ release in agreement with the results of Garcia-Lopez et al. (1996). According to stoichiometry, this increase of gH_2_ represented 32% of the total amount of [2H] saved from decreased methanogenesis, showing that at least one third of reducing equivalents spared from CH_4_ formation were not incorporated into products with a nutritional value to the host animal. Surprisingly, the increase in gH_2_ was not a consequence of greater dH_2_, which was actually lowered by 76%. Lower dH_2_ concentration may be related to the significant increase in reduced products such as caproate and heptanoate. Thus, dH_2_ may have remained at low levels because it was diverted toward reduced VFA and quickly expelled as gH_2_.

Despite the lower percentage of CH_4_ decrease in comparison to NIT and NOP, AQ had a persistent effect on fermentation as dH_2_ concentration remained lower than CON during the recovery period. Moreover, CH_4_ production was still numerically lower in AQ compared to CON after treatment removal. This outcome may be the consequence of a strong negative effect of AQ on microbiota, which may have had difficulties recovering after withdrawal. Indeed, this plant extract is known to inhibit bacterial protein synthesis by blocking the first step of RNA translation to protein in the ribosomal A site (Anke et al., 1980). Decreased bacterial activity may also account for the observed decrease in microbial protein synthesis and greater concentration of ${\text{NH}}_{4}^{+}$ during the treatment period. However, similarly to NOP, the lower ${\text{NH}}_{4}^{+}$ concentration observed with AQ during the recovery period compared with CON could be an indication of resumption of microbial activity due to greater ${\text{NH}}_{4}^{+}$ incorporation into microbial amino acids.

### How is metabolic hydrogen used when methanogenesis decreases?

#### Lost as gaseous hydrogen

With AQ and NOP, a portion of the [2H] spared from methanogenesis was lost as gH_2_. The relationship between gH_2_ and CH_4_ production seems to be treatment-dependent. Indeed, no overall correlation between variables was observed in the present study in agreement with Wang et al. (2016), whereas positive correlations were observed within treatments (CON: *r* = 0.80, *P* < 0.001; NIT: *r* = 0.54, *P* = 0.014; NOP: *r* = 0.74, *P* < 0.001; AQ: *r* = 0.67, *P* = 0.001; Supplementary Figure 1), highlighting the specificity of each methanogenesis inhibitor on rumen fermentation. Qiao et al. (2015) also observed a positive response in CH_4_ production to the replacement of headspace N_2_ with H_2_ in *in vitro* mixed batch cultures.

Gaseous H_2_ was not correlated with dH_2_ concentrations, which agrees with Wang et al. (2014) but not with Wang et al. (2016) who reported a positive and quadratic relationship between these two variables. Calculation of the supersaturation factor (S_*f*_, ratio of actual to calculated dH_2_ concentration based on gH_2_ partial pressure and Henry's law; Wang et al., 2014) indicated variable supersaturation of dH_2_ in the medium depending upon the treatment (Sf = 20.0, 77.1, 5.01, and 6.16 for CON, NIT, NOP, and AQ respectively; data not shown). The absence of equilibrium between gH_2_ and dH_2_ shows that it is not possible to use one to predict the other, and supports the complex theory of mass transfer limitation from the liquid to gaseous phase (Pauss et al., 1990). To foster a more global approach, the relationship between dissolved and gaseous H_2_ shows the interest of studying microbial biofilm structures. For instance, treatments may affect parameters involved in metabolite transfer between microorganisms, such as the diffusion coefficients, the concentration of metabolites or the distance between microbes (Leng, 2014).

Finally, it is commonly suggested that ruminal H_2_ should be maintained at low concentration to favor hydrogenase and ferredoxin reductase activity, which are two essential enzymes involved in the regeneration of reduced coenzymes produced during routine fermentation processes (Hegarty and Gerdes, 1999). With greater dissolved H_2_ concentrations, NOP contradicts this theory as substrate degradability was not modified, suggesting that the availability of oxidized coenzymes was not affected. Similarly, Guyader et al. (2015a) observed an increase of dissolved H_2_ concentrations in the rumen of cows supplemented with nitrate, without reduction of diet digestibility. Therefore, the effect of increased H_2_ concentration on ruminal fermentation requires further attention.

#### Changes in fermentation end products

Janssen (2010) suggested that [2H] consuming pathways, such as propionogenesis, would be favored with greater [2H] concentrations. However, in our study, no treatment affected propionate production (*P* = 0.219; data not shown) despite increasing H_2_ concentrations with NOP or AQ. This result may indicate that a decrease in methanogenesis would not modify glucose production through gluconeogenesis from propionate, although this effect has yet to be confirmed *in vivo*.

An increase in the average carbon chain length of end products linked to greater [2H] availability has been reported previously with the use of redox mediators as methanogenesis inhibitors (neutral red, methyl viologen, safranin O, and tannic acid; Nerdahl and Weimer, 2015). Similarly, with NOP and AQ, part of [2H] spared from methanogenesis was diverted toward the production of more energy-dense and reduced straight-chain VFA. Caproate may have been mainly produced through the propionyl-CoA pathway given that the [2H] recovery was closer to 100% with SC1.

Another alteration caused by inhibiting methanogenesis, especially with NIT, was a decrease in branched-chain VFA molar percentage. This leads to the hypothesis that amino acid fermentation was inhibited along with methanogenesis, with lower deamination of branched-chain amino acids. However, the amount of [2H] produced or consumed during protein fermentation is difficult to estimate given that several pathways with different [2H] balance can lead to the production of each amino acid (Demeyer, 1991). For instance, there can be net uptake or production of [2H] during glutamate production from glucose (Czerkawski, 1986).

The role of formate in [2H] balance still requires clarification. Leng (2014) stated that formate would be favored as electron carrier in homogenized and aqueous environments whereas H_2_ would be more efficient in dense aggregates such as in the ruminal environment. It was estimated that formate may act as an alternative electron carrier accounting for 10–20% of [2H] spared from CH_4_ formation (Ungerfeld, 2015) and may be used as a regulator of [2H] balance (Shi et al., 1997). However, despite major modifications in [2H] flow, formate concentration was not affected when inhibiting methanogenesis in the present study. In contrast, an accumulation of formate was reported *in vitro* when using alternative electron sinks to inhibit ruminal methanogenesis (Ungerfeld et al., 2003). Using lactating dairy cows supplemented with up to 21 g nitrate/kg DM, Olijhoek et al. (2016) also reported an increase in formate from 0.08 mol/100 mol VFA up to 0.45 mol/100 mol VFA. Thus, further study of the response of formate concentration in relation to methanogenesis inhibition may help to understand the role of this rumen fermentation intermediate.

#### Structural element for microbial biomass synthesis

Greater efficiency of microbial protein synthesis with NIT raises the question of the role of microbial biomass as an alternative [2H] sink. The principal component analysis revealed a negative correlation between E_MPS_ and reduced VFA, suggesting that these pathways compete for [2H] spared from CH_4_ production. Mills et al. (2001) estimated that 0.41 moles [2H] would be used per gram of microbial matter produced growing on NPN, which is close to the conditions of the present work. Then, assuming a constant microbial CP content of 54.5 g per gram of dry cell (Reichl and Baldwin, 1975) and a N content in microbial protein of 16%, daily [2H] used for microbial biomass synthesis would average 1.62, 1.89, 1.48, and 1.41 moles for CON, NIT, NOP, and AQ, respectively, representing 2.91, 3.63, 3.16, and 2.78% of [2H] produced (SC1) for the respective treatments. These percentages are in the range of previous estimations (Mills et al., 2001), and support previous suggestions that microbial biomass makes a relatively small contribution to [2H] sinks (Czerkawski, 1986; Mills et al., 2001).

That said, possible changes in microbial composition as a consequence of modified [2H] availability have not been taken into account in previous calculations, and it is possible that this could increase the importance of microbial biomass as an alternative [2H] sink. Indeed, an increase in [2H] availability as observed with NOP and AQ may increase the ratio of saturated to unsaturated microbial fatty acids in cells membrane. As proposed by Ungerfeld (2015), further studies should explore the effect of methanogenesis inhibition on microbial biomass amount and composition. Analysis of changes in the microbial community composition induced by methanogenesis inhibition using molecular techniques is also important (Denman et al., 2015; Martinez-Fernandez et al., 2016).

## Conclusions

Nitrate, nitrooxypropanol, and anthraquinone differently affected the fate of [2H] spared from decreased methanogenesis. Through its role as an electron acceptor, nitrate mostly decreased [2H] availability. Nitrooxypropanol and anthraquinone increased the amount of reduced VFA, but this was not sufficient to incorporate all extra [2H] available. As a consequence, gH_2_ increased as a way of evacuating the excess [2H]. Further work is needed to examine ways of efficiently utilizing energy spared from methanogenesis to improve animal performance. Moreover, as [2H] recovery was lower than 60% with CON, NOP, and AQ, future studies should aim at understanding the modifications in metabolic pathways (such as caproate formation) and microbial fatty acid composition resulting from CH_4_ decrease in order to improve our understanding of [2H] production and utilization. From a practical point of view, this study also showed the importance of a daily supplementation of NIT, NOP, and AQ to maintain a sustained reduction in CH_4_, despite some persistent modifications in VFA profile after removal of the inhibitors.

## Author contributions

JG wrote the experimental protocol, ran the experiment including lab analyses, statistically analyzed the data and wrote the paper. EU helped to write the experimental protocol including planning and use of the RUSITEC, and to interprete the data. In addition, EU corrected the manuscript several times. KB helped to write and validate the experimental protocol. She also helped to interprete the data and she revised the manuscript several times too. Moreover, KB provided all the equipment and staff required to run the experiment.

### Conflict of interest statement

The authors declare that the research was conducted in the absence of any commercial or financial relationships that could be construed as a potential conflict of interest.
