# Supplementary material for: Redirection of Metabolic Hydrogen by Inhibiting Methanogenesis in the Rumen Simulation Technique (RUSITEC)
Source: Front Microbiol. 2017 Mar 14;8:393. doi: 10.3389/fmicb.2017.00393 (PMC5349286; doi:10.3389/fmicb.2017.00393)
Supplement: Supplementary file 1 [file Table1.docx]

**Supplementary Material**

Table 1: Correlation matrix between rumen fermentation variables affected by methane inhibitors using a rumen simulation technique. Statistical levels were adjusted with the false discovery rate option.

| Item^1^ | Dissolved H_2_ (μM) | Total gas (mL/d) | CH_4_ (mL/d) | H_2_ (mL/d) | CO_2_ (mL/d) | N_2_O (mL/d) | dOM (%) | E_MPS_^2^ | Total VFA (mM) | Acetate (mM) | Propionate (mM) |
| --- | --- | --- | --- | --- | --- | --- | --- | --- | --- | --- | --- |
| Total gas (mL/d) | -0.295 |  |  |  |  |  |  |  |  |  |  |
| CH_4_ (mL/d) | -0.195 | 0.506^*^ |  |  |  |  |  |  |  |  |  |
| H_2_ (mL/d) | -0.232 | 0.123 | -0.215 |  |  |  |  |  |  |  |  |
| CO_2_ (mL/d) | 0.023 | -0.539^*^ | -0.762^***^ | -0.469 |  |  |  |  |  |  |  |
| N_2_O (mL/d) | -0.103 | -0.470 | -0.575^*^ | -0.475 | 0.834^***^ |  |  |  |  |  |  |
| dOM (%) | 0.200 | 0.269 | 0.102 | 0.288 | -0.283 | -0.216 |  |  |  |  |  |
| E_MPS_^2^ | 0.067 | -0.511^*^ | -0.347 | -0.597^**^ | 0.709^**^ | 0.800^***^ | -0.376 |  |  |  |  |
| Total VFA (mM) | -0.077 | 0.498^*^ | 0.519^*^ | 0.290 | -0.663^**^ | -0.295 | 0.689^**^ | -0.454 |  |  |  |
| Acetate (mM) | -0.225 | -0.018 | 0.173 | -0.532^*^ | 0.196 | 0.508^*^ | 0.343 | 0.388 | 0.459 |  |  |
| Propionate (mM) | 0.227 | 0.309 | 0.216 | 0.440 | -0.488 | -0.210 | 0.800^***^ | -0.346 | 0.905^***^ | 0.405 |  |
| Isobutyrate (mM) | 0.257 | 0.517^*^ | 0.606^**^ | 0.427 | -0.832^***^ | -0.587^**^ | 0.640^**^ | -0.552^*^ | 0.877^***^ | 0.120 | 0.837^***^ |
| Butyrate (mM) | 0.049 | 0.472 | 0.355 | 0.597^**^ | -0.717^***^ | -0.469 | 0.748^***^ | -0.635^**^ | 0.921^***^ | 0.155 | 0.906^***^ |
| Valerate (mM) | 0.376 | 0.339 | 0.119 | 0.814^***^ | -0.647^**^ | -0.499^*^ | 0.601^**^ | -0.638^**^ | 0.689^**^ | -0.196 | 0.794^***^ |
| Isovalerate (mM) | 0.080 | 0.679^**^ | 0.769^***^ | 0.365 | -0.938^***^ | -0.786^***^ | 0.410 | -0.700^**^ | 0.721^***^ | -0.118 | 0.564^*^ |
| Caproate (mM) | -0.284 | 0.514^*^ | 0.426 | 0.562^*^ | -0.758^***^ | -0.635^**^ | 0.011 | -0.737^***^ | 0.395 | -0.494 | 0.175 |
| Heptanoate (mM) | -0.284 | 0.514^*^ | 0.426 | 0.563^*^ | -0.758^***^ | -0.636^**^ | 0.012 | -0.737^***^ | 0.395 | -0.494 | 0.175 |
| Ethanol (mM) | 0.301 | -0.565^*^ | -0.705^**^ | -0.110 | 0.711^**^ | 0.547^*^ | 0.129 | 0.561^*^ | -0.360 | 0.285 | -0.032 |
| NH_4_^+^ (mM) | -0.035 | 0.428 | 0.122 | 0.834^***^ | -0.663^**^ | -0.655^**^ | 0.308 | -0.835^***^ | 0.441 | -0.513^*^ | 0.400 |
| Formate (mM) | 0.365 | 0.058 | 0.311 | 0.596^**^ | -0.676^**^ | -0.692^**^ | 0.138 | -0.573^*^ | 0.196 | -0.576^*^ | 0.206 |
| Propanol (mM) | 0.585^*^ | -0.021 | -0.353 | 0.760^***^ | -0.185 | -0.202 | 0.546^*^ | -0.256 | 0.303 | -0.184 | 0.615^**^ |

^1^ ^*^ *P* ≤ 0.10, ^**^ *P* ≤ 0.05, ^***^ *P* ≤ 0.01

^2^ Efficiency of microbial protein synthesis expressed as grams microbial N produced per kilogram OM fermented

Table 1 (end): Correlation matrix between rumen fermentation variables affected by methane inhibitors using a rumen simulation technique. Statistical levels were adjusted with the false discovery rate option.

| Item^1^ | Isobutyrate (mM) | Butyrate (mM) | Valerate (mM) | Isovalerate (mM) | Caproate (mM) | Heptanoate (mM) | Ethanol (mM) | NH_4_^+^ (mM) | Formate (mM) |
| --- | --- | --- | --- | --- | --- | --- | --- | --- | --- |
| Butyrate (mM) | 0.900^***^ |  |  |  |  |  |  |  |  |
| Valerate (mM) | 0.813^***^ | 0.870^***^ |  |  |  |  |  |  |  |
| Isovalerate (mM) | 0.901^***^ | 0.748^***^ | 0.670^**^ |  |  |  |  |  |  |
| Caproate (mM) | 0.473 | 0.515^*^ | 0.546^*^ | 0.683^**^ |  |  |  |  |  |
| Heptanoate (mM) | 0.473 | 0.516^*^ | 0.547^*^ | 0.683^**^ | 1.000^***^ |  |  |  |  |
| Ethanol (mM) | -0.439 | -0.316 | -0.269 | -0.719^***^ | -0.856^***^ | -0.856^***^ |  |  |  |
| NH_4_^+^ (mM) | 0.546^*^ | 0.696^**^ | 0.771^***^ | 0.613^**^ | 0.838^***^ | 0.838^***^ | -0.516^*^ |  |  |
| Formate (mM) | 0.494 | 0.383 | 0.630^**^ | 0.559^*^ | 0.593^**^ | 0.593^**^ | -0.428 | 0.552^*^ |  |
| Propanol (mM) | 0.445 | 0.550^*^ | 0.746^***^ | 0.182 | 0.037 | 0.038 | 0.353 | 0.456 | 0.375 |

^1^ ^*^ *P* ≤ 0.10, ^**^ *P* ≤ 0.05, ^***^ *P* ≤ 0.01

Figure 1: In vitro (rumen simulation technique) effect of a control treatment (CON) and nitrate (NIT), 3-nitrooxypropanol (NOP) and anthraquinone (AQ) on the relationship between gaseous H_2_ and CH_4_ production.
